# Association of right atrium and sinoatrial node irradiation with atrial fibrillation and radiation-induced heart disease in non-small cell lung cancer

**DOI:** 10.2340/1651-226X.2026.43885

**Published:** 2026-02-06

**Authors:** Christoffer Sander Graven-Nielsen, Anna Jakobsen Kragh, Rasmus Froberg Brøndum, Marie Louise Milo, Martin Skovmos Nielsen, Asbjørn Ettrup-Christensen, Kasper Lind Laursen, Weronika Maria Szejniuk

**Affiliations:** a Department of Clinical Medicine, Aalborg University, Aalborg, Denmark; b Department of Oncology & Clinical Cancer Research Centre, Aalborg University Hospital, Aalborg, Denmark; c Centre for Clinical Data Science, Aalborg University & Aalborg University Hospital, Aalborg, Denmark; d Department of Medical Physics, Aalborg University Hospital, Aalborg, Denmark; e Department of Cardiology, Aalborg University Hospital, Aalborg, Denmark

**Keywords:** Non-small-cell lung, radiotherapy, cardiac toxicity, atrial fibrillation, sinoatrial node, organs at risk, thoracic radiation, right atrium, radiation-induced heart disease

## Abstract

**Background and purpose:**

Radiotherapy (RT) is a key treatment for locally advanced non-small cell lung cancer (NSCLC). Tumours near the heart may result in unintended cardiac radiation exposure, increasing the risk of cardiotoxicity, such as de novo atrial fibrillation (DNAF) and de novo heart diseases (DNHD) as ischemic heart disease, heart failure, arterial hypertension or sudden cardiac death. This study investigated associations between radiation dose to cardiac substructures and risk of DNAF and DNHD.

**Patient/material and methods:**

This retrospective cohort study included patients treated between January 1, 2010, and December 31, 2020 for NSCLC with definitive RT. The heart, right atrium (RA) and sinoatrial node (SAN) were delineated. Associations between dose-volume parameters and cardiac outcomes were analysed using multivariable models adjusted for relevant confounders. Kaplan-Meier curves estimated survival; *p*-values < 0.05 were significant.

**Results:**

Among 273 included patients, 9.5% had AF pre-RT and 12.8% developed DNAF. DNAF was significantly associated with SAN D_max_ (hazard ratio [HR] = 1.01), RA D_max_ (HR = 1.02), RA D_mean_ (HR = 1.03), mean heart dose (MHD) (HR = 1.04) and heart V40Gy (HR = 1.03). One-year probabilities of DNAF and DNHD were 9.3% and 11%, increasing to 12.2% and 13.2% at 2 years. DNHD was significantly associated with SAN D_max_ (HR = 1.02), RA D_max_ (HR = 1.02), RA D_mean_ (HR = 1.04), MHD (HR = 1.06), heart V25Gy (HR = 1.03) and V40Gy (HR = 1.03).

**Interpretation:**

The RA and SAN may be considered organs at risk in future RT planning. Minimising cardiac radiation is important to reduce DNAF and DNHD risk. Validation in an independent cohort is warranted.

## Introduction

Radiation therapy (RT) is a cornerstone of cancer treatment and is often combined with chemotherapy and surgery. In patients with non-small cell lung cancer (NSCLC), the proximity of tumours to the heart results in a risk of radiation exposure to nearby cardiac structures, potentially resulting in cardiotoxicity [1]. The sinoatrial node (SAN) is the heart’s primary pacemaker [2] and may receive high doses of radiation in the case of tumours located in the upper lung regions. Similarly, the right atrium (RA), which is localised near the SAN, is also at risk of radiation exposure. Damage to these structures may lead to radiation-induced arrhythmias, such as de novo atrial fibrillation (DNAF), and other de novo heart diseases (DNHD), including arterial hypertension, unstable angina, coronary artery disease, acute myocardial infarction, heart failure or sudden cardiac death [3].

Atrial fibrillation (AF) is the most prevalent cardiac arrhythmia [4] and is associated with a higher risk of stroke, heart failure [5, 6], decreased quality of life and increased mortality [7] in cancer survivors [8]. Besides RT-induced cardiotoxicity, other unmodifiable and modifiable risk factors can have a major impact on the development of AF in patients treated for NSCLC, such as age, sex, arterial hypertension, smoking, chronic obstructive pulmonary disease, physical activity, diabetes and obesity [9, 10].

Only a limited number of studies have investigated the relationship between maximum radiation doses to the RA [11] and other cardiac substructures [12–15] and the risk of developing cardiac toxicity, including DNAF. Nevertheless, further research is needed to improve our understanding of the mechanisms that contribute to this risk.

The primary aim of this study was to evaluate the association between radiation exposure to the SAN and RA and the risk of DNAF in patients treated curatively for NSCLC. Then, the incidence of DNHD and survival in relation to both DNAF and DNHD were evaluated.

## Patients/material and methods

### Patient data

This retrospective cohort study included consecutive patients diagnosed with NSCLC and treated with curative RT of 60–66 Gy in 30–33 fractions concomitant with chemotherapy, consisting of a platinum-based doublet with vinorelbine, between the 1^st^ of January 2010 and 31^st^ of December 2020 at the Department of Oncology, Aalborg University Hospital, Denmark. The treatment techniques were either 3D conformal RT with field modulation as field-in-field or wedges or intensity-modulated RT (IMRT) with sliding window. All plans were calculated using Anisotropic Analytical Algorithm (AAA). 38 patients were excluded due to non-NSCLC diagnosis (*n* = 9), postoperative RT (*n* = 13), metastatic disease before RT (*n* = 8) or interruption or discontinuation of RT (*n* = 8). Consequently, the final cohort consisted of 273 patients.

Descriptive data were obtained through a review of patients’ electronic health records. Study data were collected and managed using REDCap electronic data capture tools hosted at Aalborg University Hospital [16, 17]. Patient-related data included age, sex, comorbidities using a modified Charlson comorbidity index without lung cancer [18], smoking, pre- and post-RT cardiac diseases, pre-RT haemoglobin (Hgb) levels and survival data. Lung cancer-related data were histopathological type, TNM (6^th^, 7^th^ and 8^th^ edition) [19–21] and treatment-related data. The cardiotoxicity data were collected through a review of medical records before and after lung cancer RT and consisted of cardiac conditions prior to RT and post RT, development of DNAF and DNHD (de novo ischemic heart disease, including unstable angina, coronary artery disease, acute myocardial infarction, de novo heart failure and de novo arterial hypertension). The patients were followed until progression, other malignancy or death.

### Dose-volume data

**Figure 1:**
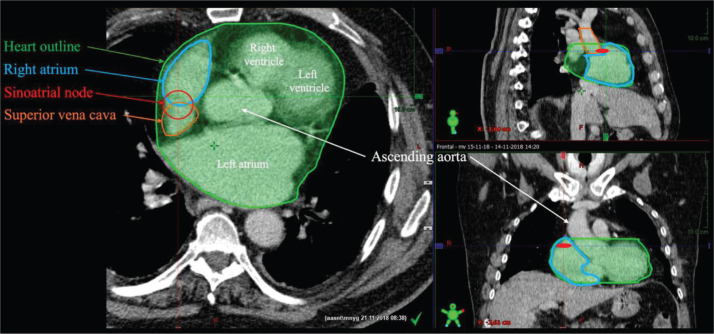
Example of delineation of the heart, sinoatrial node and right atrium on a planning RT scan. Provided by study investigators.

The SAN and the RA were delineated on RT planning CT scans, with a slice thickness of 2.0 or 2.5mm, using the ARIA®Ver. 18.0 [22–23] (Figure 1). The SAN was delineated manually according to RT planning delineation atlas [21]. Delineation of the RA was likewise performed manually according to established national guidelines on delineation of cardiac structures [24]. Delineation of the heart was performed as a part of the clinical RT planning. The extracted dosimetric data were the mean heart dose (MHD), the volume of the heart in percentage that received >25 Gy (V_25Gy_) and >40 Gy (V_40Gy_). For the SAN and RA segmentations, the extracted dose–volume values were mean (D_mean_) and maximum doses and (D_max_), defined as radiation dose to the 1 cubic centimeter (cc) volume (D1cc) for the RA and 0.1cc for the SAN (D0.1cc).

### Intra- and Inter-observer variability of dose-volume data

The delineation was performed by two independent investigators (investigators A and B). Thus, data quality was evaluated through an assessment of inter- and intra-observer variability. A randomly selected subset of 10 RT planning CT scans from the cohort was used, and both investigators delineated the SAN and RA to determine inter-observer variability. The same 10 CT scans were used to delineate the SAN and RA by each investigator with a 1-week interval to assess intra-observer variability. Inter-observer variability was quantified by calculating the intraclass coefficient correlation (ICC) in RT doses of individual heart structures between investigators A and B. Intra-observer variability was measured by ICC in RT doses of individual heart structures in the delineations performed by investigator A in two separate sessions, with the same process applied to investigator B.

The SAN and RA delineations were validated by a cardiologist specialising in heart-CT and a thoracic oncologist, confirming the accuracy of the SAN and RA delineation. Based on these validations, the median and maximum doses to the heart, SAN and RA in all patients were considered reliably collected for further analysis.

### Statistical methods

The baseline characteristics between the two groups were compared using a *t*-test for continuous data and Fisher’s exact test for categorical data. Age was calculated from the date of birth to the date of RT-start. The reliability of the dose-volume data was evaluated by conducting ICC to assess the inter- and intra- observer variability.

Time to event, including time to DNAF and DNHD, was calculated from the first day of RT to the date of event or death. Thus, median follow-up and overall survival were calculated from the first day of RT to death or censoring, and time to event was calculated from the first day of RT to the event with death as a competing risk. Observations were censored at the date of data entry if no other events had been observed. To perform the primary DNAF analysis, all patients with pre-RT AF were excluded, and the cohort was subdivided into a group with post-RT AF and a group with no post-RT AF. Likewise, to perform the secondary DNHD analysis, all patients with pre-RT cardiac disease were excluded, and the cohort was subdivided into a group with DNHD and a group with no DNHD.

A multivariable Cox regression analysis was conducted to calculate the cause-specific hazard ratio (HR) for the occurrence of DNAF or DNHD based on cardiac dose variables, adjusted for clinically relevant confounders such as age and sex. In the DNAF analyses, pre-RT arterial hypertension and pre-RT Hgb levels were further adjusted for. The competing risk analysis for each of the dose-volume variables was plotted using a cumulative incidence function. Kaplan-Meier survival curves were performed to estimate overall survival. Survival was calculated from the start of RT to death for overall survival and for DNAF and DNHD from the date of the given post-RT cardiac diagnosis to death. Statistical analyses were conducted using STATA software version 18 and R software 4.4.2, and significance was set at a two-sided *p*-value of < 0.05. Continuous variables were noted with medians (med) and interquartile range (IQR). Categorical variables were noted as frequency (*n*) and percentages (%). The project was approved by the National Committee on Health Research Ethics (reg. no 2200994) and reported to the North Denmark Region (F2022-027).

## Results

### Patient data

Out of the 273 patients, 26 patients (9.5%) were diagnosed with pre-existing AF and were further excluded from the cohort leading to final number of 247 patients. The cohort was divided into post-RT DNAF (*n* = 35) and no post-RT DNAF (*n* = 212) (Table 1). Significant differences between the groups were observed, with higher age (*P* = 0.004), a higher proportion of 60 Gy/30 fx compared to 66 Gy/33 fx (*P* = 0.013) and a higher incidence of de novo ischaemic heart diseases and de novo heart failure (*P* = 0.001) in the DNAF group compared to the non-DNAF group. DNAF was neither associated with NSCLC progression (*P* = 1.00) nor use of palliative systemic treatment (*P* = 1.00).

**Table 1:** Frequency (%) of the baseline demographics, clinical characteristics and significant differences.

Demographics	Total cohort *n* (%)	No pre-RT AF & DNAF *n* (%)	DNAF *n* (%)	*P*-value
Number of patients	247	212	35	
Median age [IQR]	68 (62–72)	67.1 (61–72)	70.7 (64–74)	0.004
Male	151 (61.1)	127 (59.9)	24 (68.6)	0.356
Performance status				
0	130 (52.6)	110 (51.9)	20 (57.1)	0.926
1	100 (40.5)	87 (41.0%)	13 (37.1)	
2	16 (6.5)	14 (6.6)	2 (5.7)	
3	1 (0.4)	1 (0.5)	0 (0)	
Tobacco ever used	238 (96.4)	205 (96.7)	33 (94.3)	0.619
Pack-years [IQR]	25–50	27.5–50	23–50	0.690
**Comorbidities pre-RT**				
Charlson’s comorbidity index				
0	106 (42.9)	94 (44.3)	12 (34.3)	0.469
1	90 (36.4)	74 (35.8)	14 (40.0)	
≥ 2	51 (20.7)	42 (19.8)	9 (25.7)	
COPD [%]	69 (27.9)	56 (26.4)	13 (37.1)	0.583
Peripheral vascular disease	30 (12.5)	26 (12.3)	4 (11.4)	
Diabetes mellitus	26 (10.5)	19 (9.0)	7 (20.0)	
Cerebrovascular accident	18 (7.3)	16 (7.5)	2 (5.7)	
Liver disease	6 (2.4)	6 (2.8)	0 (0)	
Leukaemia or lymphoma	5 (2)	4 (1.9)	1 (2.9)	
No comorbidities	107 (43.3)	94 (44.3)	13 (37.1)	0.466
**NSCLC tumour characteristics**				
I	11 (4.5)	11 (5.2)	0 (0)	0.889
II	32 (13)	27 (3.8)	5 (14.3)	
IIIA	131 (53)	110 (51.9)	21 (60.00)	
IIIB	69 (27.9)	60 (28.3)	9 (25.71)	
IIIC	4 (1.6)	4 (1.9)	0 (0)	
Tumour size [IQR mm]	28–60	25–60	33–60	0.885
Location				
Right lung	150 (60.7)	126 (59.4)	24 (68.6)	0.219
Left lung	94 (38.1)	84 (39.6)	10 (28.6)	
Mediastinum	3 (1.2)	2 (0.9)	1 (2.9)	
TNM lymph nodes				
N0	54 (21.9)	50 (23.6)	4 (11.4)	0.0946
N+	193 (78.1)	162 (76.4)	31 (88.6)	
**Systemic treatment**				
Carboplatin/Vinorelbine	227 (91.9)	193 (91.0)	35 (100)	0.225
Other	12 (4.9)	12 (5.7)	0 (0)	
Missing/none	8 (3.2)	7 (3.3)	1 (2.9)	
RT dose				
66 Gy/33 fx	156 (63.2)	141 (66.5)	15 (42.9)	0.013
60 Gy/30 fx	91 (36.8)	71 (33.5)	20 (57.1)	
RT type				
3D conformal RT	182 (73.7)	153 (72.2)	29 (82.9)	0.218
IMRT	65 (26.3)	59 (27.8)	6 (17.1)	
Hgb (mmol/l) status pre-RT				
Median [IQR]	7.8 [4.90, 10.2]	7.8 [4.90, 10.2]	7.8 [5.20, 9.30]	0.888
Mean (SD)	7.62 (0.993)	7.7 (0.987)	7.68 (0.887)	
**Cardiac status pre-RT**				
Atrial firbillation	0 (0)	0 (0)	0 (0)	
Arterial hypertension	102 (41.3)	84 (39.6)	18 (51.4)	0.588
Ischaemic heart disease	32 (13.0)	28 (13.2)	4 (11.4)	
Heart failure	3 (1.2)	2 (0.9)	2 (5.7)	
Total cardiac status pre-RT	115 (46.6)	98 (46.2)	18 (51.4)	
**Cardiac status post-RT**				
De novo atrial fibrillation	35 (14.2)	0 (0)	35 (100)	
De novo arterial hypertension	5 (2)	5 (2.4)	0 (0)	0.001
De novo ischaemic heart disease	7 (2.8)	2 (0.9)	5 (14.3)	
De novo heart failure	12 (4.9)	5 (2.4)	7 (20.0)	
Total radiation-induced heart disease	46 (18.6)	12 (5.7)	35 (100)	

RT: radiotherapy; AF: Atrial fibrillation; DNAF= De novo atrial fibrillation; NSCLC: Non-small cell lung cancer; Hgb: haemoglobin; n: number of patients; IQR: Interquartile range; TNM=6th-8th edition; 3D: three-dimensional; IMRT: Intensity-modulated radiation therapy.

The DNHD group (*n* = 19) showed significant differences similar to those seen in the DNAF subgroup, including higher age (*P* = 0.005) and lower RT dose (*P* = 0.03).

The incidence of de DNAF post-RT was 12.8%, whereas the prevalence was 9.5% pre-RT. Post-RT incidence of de novo arterial hypertension was 1.8% and 3.7% for de novo ischaemic heart disease, compared to a pre-RT prevalence of 44% and 13.6%, respectively. The incidence of de novo heart failure post-RT was 4.4%, while the prevalence of heart failure pre-RT was 1.8%.

### Dose-volume data

The median of the MHD was 7.3 Gy (IQR: 2.7–15.0), V_25Gy_ of the heart was 8.6% (IQR: 2.0–21.5) and V_40Gy_ of the heart was 4.5% (IQR: 0.7–10.6). The median D_mean_ to RA was 5.1 Gy (IQR: 1.5–17.3) with a median D_max_ of 31.8 Gy (IQR: 6.8–53.6). For SAN, the median D_max_ was 14.0 Gy (IQR: 3.0–46.8).

### Inter- and intra-variability of dose-volume outcomes

The degree of consistency and reliability between the two investigators was reflected in interobserver variability ICC values (0.998–0.768) and intraobserver variability ICC values (0.995–0.673) (Table 2).

**Table 2:** Interobserver variability and intra-individual variability with ICC (*n* = 10).

	Investigator A [mean (SD)]	Investigator B [mean (SD)]	Interobserver variability [ICC]
D_max_ SAN, day 1 [Gy]	31.2 (26.0)	31.5 (25.7)	0.998
D_max_ SAN, day 8 [Gy]	32.9 (25.4)	32.3 (25.6)	0.998
**Intraobserver variability [ICC]**	0.987	0.995	
V_RA_, day 1 [cm^3^]	96.8 (12.5)	93.4 (10.7)	0.768
V_RA_, day 8 [cm^3^]	89.1 (14.1)	96.5 (14.0)	0.768
**Intraobserver variability [ICC]**	0.692	0.673	
D_mean_ RA, day 1 [Gy]	13.7 (15.0)	13.9 (15.0)	0.997
D_mean_ RA, day 8 [Gy]	13.9 (15.2)	14.1 (15.4)	0.998
**Intraobserver variability [ICC]**	0.999	0.995	

Max: maximum; SAN: sinoatrial node; RA: right atrium; ICC: intraclass correlation coefficient.

### Time to cardiac events

**Figure 2:**
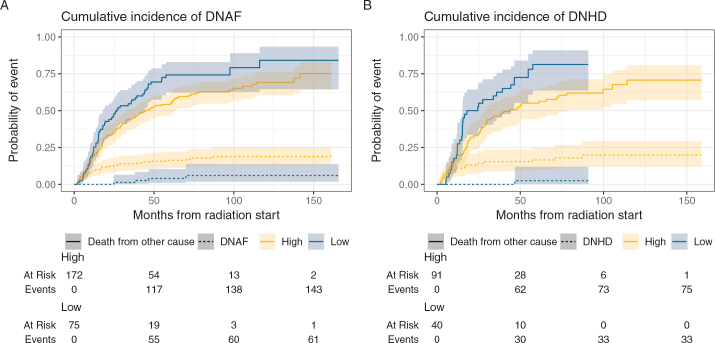
Cumulative incidence functions (CIF) of DNAF (A) and DNHD (B) event and exposure to mean RA.

The median observation time was 27.4 months (IQR: 12.5–57.8) from the start of RT to cardiac event, death or last observation. The median time from start of RT to DNAF was 16 months (IQR: 6.4–39.4), the median time to de novo ischemic heart disease was 10 months (IQR: 6.8–5.2) and the median time to de novo heart failure was 30.4 months (IQR: 6.8–74). There was a 9.3% probability of DNAF and a 11% probability of DNHD at 12 months, while the probability of death from other causes during the same period was 19.8% for both groups. At 24 months, the probability of DNAF was 12.2%, DNHD was 13.2% and death from other causes was 37.2% (Figure 2).

### Risk of cardiac events

A significant association with an increased risk of DNAF was observed for both SAN and RA, consisting of D_max_ to the SAN (HR: 1.014, 95% confidence interval [CI]: 1.000–1–029), D_max_ to the RA (HR: 1.017, 95% CI: 1.002–1.032), the D_mean_ to the RA (HR: 1.031, 95% CI: 1.007–1.056), MHD (HR: 1.040, 95% CI: 1.006–1.076) and V_40Gy_ (HR: 1.024, 95% CI: 1.000–1.048). Age was a significant confounding variable, showing an association with increased risk of DNAF (Table 3) and DNHD (Table 4) for all dose-volume variables with different degrees of risk. Pre-existing arterial hypertension, pre-RT Hgb levels or male sex showed no significant association with the risk of developing DNAF (Table 3). All dose-volume variables were significantly associated with an increased risk of DNHD (Table 4).

**Table 3:** Cox-regression analyses for radiation exposures and confounders for de novo atrial fibrillation.

Variables	Hazard ratio	*P*-value	95% confidence interval
D_max_ SAN	1.014	0.041*	1.000–1.029
Age	1.061	0.019*	1.009–1.115
Male	1.850	0.110	0.869–3.938
Arterial hypertension pre-RT	1.115	0.756	0.559–2.225
Pre-RT Hgb	0.837	0.323	0.589–1.190
D_mean_ RA	1.031	0.011*	1.007–1.056
Age	1.061	0.020*	1.009–1.115
Male	1.932	0.091	0.899–4.153
Arterial hypertension pre-RT	1.205	0.599	0.600–2.416
Pre-RT Hgb	0.858	0.409	0.596–1.234
D_max_ RA	1.017	0.026*	1.002–1.032
Age	1.066	0.012*	1.014–1.120
Male	1.784	0.129	0.843–3.771
Arterial hypertension pre-RT	1.157	0.678	0.581–2.302
Pre-RT Hgb	0.828	0.291	0.584–1.175
MHD	1.040	0.021*	1.006–1.076
Age	1.056	0.028*	1.006–1.109
Male	1.729	0.151	0.819–3.649
Arterial hypertension pre-RT	1.170	0.657	0.584–2.343
Pre-RT Hgb	0.842	0.354	0.585–1.212
V_25Gy_	1.017	0.053	0.999–1.035
Age	1.055	0.032*	1.004–1.107
Male	1.740	0.146	0.825–3.671
Arterial hypertension pre-RT	1.177	0.645	0.587–2.359
Pre-RT Hgb	0.844	0.358	0.588–1.212
V_40Gy_	1.024	0.044*	1.000–1.048
Age	1.055	0.033*	1.004–1.108
Male	1.713	0.158	0.811–3.617
Arterial hypertension pre-RT	1.179	0.642	0.588–2.364
Pre-RT Hgb	0.851	0.385	0.591–1.224

RT: radiotherapy; AF: atrial fibrillation; Max: maximum; SAN: sinoatrial node; RA: right atrium; Hbg: Haemoglobin; V_25Gy_: % of heart receiving > 25Gy; V_40Gy_: % of heart receiving > 40Gy;

* : statistically significant.

**Table 4:** Cox-regression analyses for radiation exposures and confounders for de novo heart diseases.

Variables	Hazard ratio	*P*-value	95% confidence interval
D_max_ SAN	1.020	0.036*	1.001–1.040
Age	1.102	0.008*	1.025–1.185
Male	1.915	0.222	0.676–5.426
D_mean_ RA	1.037	0.015*	1.007–1.068
Age	1.108	0.006*	1.029–1.192
Male	1.915	0.215	0.685–5.352
D_max_ RA	1.022	0.038*	1.001–1.044
Age	1.105	0.006*	1.028–1.187
Male	1.726	0.296	0.620–4.799
MHD	1.058	0.015*	1.011–1.106
Age	1.109	0.005*	1.031–1.192
Male	1.786	0.258	0.654–4.880
V_25Gy_	1.027	0.020*	1.004–1.050
Age	1.107	0.005*	1.031–1.189
Male	1.900	0.213	0.692–5.189
V_40Gy_	1.034	0.015*	1.006–1.062
Age	1.107	0.007*	1.028–1.192
Male	1.694	0.309	0.613–4.676

RT: radiotherapy; AF: atrial fibrillation; Max: maximum; SAN: sinoatrial node; RA: right atrium; V_25Gy_: % of heart receiving > 25Gy; V_40Gy_: % of heart receiving > 40Gy;

* : statistically significant.

### Survival

**Figure 3:**
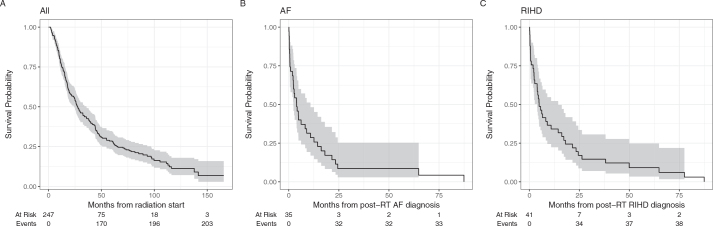
Survival in the whole cohort from the radiation therapy start (A), from the DNAF diagnosis (B) and from the DNHD diagnosis (C).

A total of 203 (82.2%) patients died during the observation time. The median overall survival was 27.4 months (IQR: 24.5–35.7) with an IQR of 11–43 months. Only 30 patients (12.1%) received immunotherapy with or without chemotherapy, either as first-line or subsequent lines of palliative treatment following progression. Of the 82% of patients who died, 48% of deaths were due to NSCLC progression (Table S1). The median overall survival in patients with DNAF was 3.9 months (IQR: 2.3–10.9), and for patients with DNHD, it was 4.9 months (IQR: 3.9–14.4) (Figure 3).

## Discussion and conclusion

The study showed a significant association between radiation exposure to the RA, SAN, MHD and V_40Gy_ to the heart, with an increased risk of DNAF and DNHD.

The results of the study may suggest that the RA and SAN could be considered as organs at risk during RT treatment of NSCLC patients. Furthermore, the current clinical practice of dose constraints for V_40Gy_ and MHD confirms the association between these variables and the increased risk of DNAF and DNHD and should be further practised. In the current study, Dmax to the SAN was defined as D0.1cc, which has a greater robustness as a metric than Dmax to any single voxel. Whereas Dmax reflects a single, isolated hot spot, D0.1cc represents the average high dose delivered to a small but meaningful tissue volume, making it a more reliable indicator of potential toxicity. Nevertheless, the small size of the SAN on CT scans, necessitating delineation based on anatomical localisation rather than direct visual confirmation can potentially lead to variability in delineation among different practitioners. Additionally, the SAN’s small volume can lead to position shift across the RT fractions, introducing uncertainty in radiation dose delivery.

Notably, the lower median survival rates observed in patients with DNAF and DNHD compared to the overall median survival may indicate that the development of these conditions significantly increases mortality, underscoring the critical need for preventive measures. However, it is important to note that these survival rates are calculated from the time of diagnosis, making them inherently lower than overall survival rates, which are calculated from the start of RT. Furthermore, common risk factors for the development of heart disease were investigated as confounding variables. This revealed age as an exacerbating factor in the development of DNAF and DNHD. Since the approval of consolidation immunotherapy was issued in Denmark in 2019, limited to stage III with PD-L1 expression ≥ 25% [25], less than 5% of patients received consolidation immunotherapy. Consequently, use of immunotherapy was not considered a significant factor increasing the risk of DNAF.

The association between dose to the RA and increased risk of DNAF found in this study is consistent with the findings of a previous study by Kim et al. [11]. However, they also found a significant association between D_max_ > 20 Gy to the SAN and development of DNAF, which was not supported by the results of our study. This discrepancy may be explained by the fact that patients with pre-RT AF were not excluded from their study. The study also found no predictive value of SAN D_max_ for the development of non-AF cardiac events. This contrasts with the results of our study, showing a significant association between SAN and DNHD. It is important to note that the prevalence of pre-RT AF in this study was higher (9.5%) compared to 2.5%, potentially due to the higher prevalence of AF among Caucasians compared to East Asians [10]. Another study by Walls et al. [12] investigated AF onset and reported an incidence of 6% for DNAF, all classified as grade 3–4, showing a strong correlation with dose metrics for both the left and right pulmonary vein. Finally, Tohidinezhad et al. showed a similar prevalence (9.1%) and incidence (11.2%) of DNAF in NSCLC [13]. Similarly to our study, age was reported a predictive factor of DNAF. Although dose-volume variables investigated in their study included RA D_mean_, RA D_max,_ and SAN D_max_, the authors reported the predictive performance of dose-volume variables derived from multivariable classification models, both when considering all variables together and in conjunction with clinical variables. The authors reported a dose-volume model with a (ROC-AUC) of the dose-volume measures of cardiac substructures for predicting the new-onset AF of highest 0.61. When combined with clinical variables, the ROC-AUC showed a modest increase from 0.79 to 0.82. The D_max_ to the left atrium (LA) was identified as the most predictive factor for the development of DNAF (AUC of 0.61), comparable in scale to the ROC-AUC observed for the D_mean_ to the RA in our study. McWilliam et al. [26] investigated the association between cardiac substructure dose and outcomes, but focusing on overall survival rather than risk of DNAF. Their findings showed that survival was influenced not only by dose to specific substructures, including RA, but also by clinical factors such as PS, lymph node status and tumour volume. However, overall survival of NSCLC is heavily influenced by many non-cardiac factors and subsequent palliative treatment, making survival a complex endpoint for interpreting cardiac dose effects. Atkins et al. [27] reported that higher doses to coronary substructures, particularly the left anterior descending coronary artery, were associated with increased major adverse cardiac events and mortality. Our study investigated DNHD, while major adverse cardiac events included other cardiac endpoints than AF. Thus, the results of the mentioned study, while highly relevant, are not directly comparable to our study.

One of the major strengths of this study is its focus on associating radiation dose to specific cardiac substructures with a particular event: AF, rather than relying on composite outcomes, such as major adverse cardiac events or proxy measures like overall survival. This distinction is crucial for clarifying the heterogeneous results observed in the relationship between dose to cardiac substructures and survival, as survival is a result of multiple mechanisms of action. Furthermore, the delineations of the SAN and RA were validated by cardiologist specialising in cardiac CT and oncologist, ensuring a precise delineation. Since the study centre is a university hospital, the delineation of the heart structures adheres to international guidelines, ensuring high quality of treatment plans. Additionally, the inter- and intra-observer variability analysis demonstrated low variability in dose-volume data, thus confirming the reliability of the exposure measurements. The use of the Danish Civil Registration System (CPR) to evaluate pre- and post-RT cardiotoxicity further enhanced the data quality, as no patients were lost to follow-up. Furthermore, potential clinical confounders were addressed through multivariable Cox regression analyses, adjusting for cardiac dose-volume variables and improving the validity of the findings.

The study presents results from a consecutive cohort that is representative of the Danish population of lung cancer patients treated at the RT centre, thereby minimising the risk of selection bias. All patients had complete follow-up, as verified by the Danish National Patient Registry, with no patients lost to follow-up. However, recall bias may have influenced the results, as patients might have underreported cardiac symptoms such as palpitations or chest pain. As a result, investigating the exacerbation of AF or the progression of pre-existing heart diseases following RT was not feasible due to the retrospective nature of the study. Surveillance bias is another concern, as the reporting of cardiotoxicity may have increased over the 10 years of follow up. Additionally, some cases of AF may be undiagnosed, particularly if the AF was paroxysmal or diagnosed in primary care. A prospective study design could have mitigated some of these biases by conducting electrocardiograms at baseline to identify chronic AF cases, though paroxysmal AF would remain challenging to detect. Competing risk bias was also present, due to high mortality in NSCLC patients, as many patients died from causes such as progression of disease or infections before developing post-RT cardiotoxicity. Some clinical factors, such as weight loss, were not available in the clinical records, limiting our ability to investigate potential residual confounding factors like cachexia or treatment-related frailty. The multiple testing of dose to cardiac substructures could potentially increase the risk of false positive associations. However, the measures were correlated, making proper statistical adjustment challenging, and the fact that all associations trend in the same direction strengthens confidence in the overall finding. Although HR close to unity may suggest limited clinical relevance, this interpretation should be made in light of the exposure scale. As radiation dose was modelled per 1% increase, the estimated effects reflect very small unit changes, and larger dose contrasts may therefore have clinically meaningful implications. Finally, due to the retrospective nature of this cohort and the lack of reliable automatic delineations for all substructures of the heart, dose-volume analyses were limited to the RA and SAN. This limitation may restrict the ability to determine whether the observed associations are specific to these regions or indicative of a broader cardiac dose effect. Future prospective studies should be considered with inclusion of more comprehensive cardiac substructure analyses using standardised automated delineation techniques to facilitate more efficient and accurate evaluation of the heart’s substructures.

Despite the significant findings of this study, it remains a priority to ensure sufficient RT treatment of NSCLC regardless of tumour proximity to the heart. As observed in the present study, 48.2% of patients died due to progression of lung cancer, whereas only 6% died due to cardiac conditions. However, following this study period, survival rates among NSCLC patients have improved. This underlines the clinical relevance of increased focus on adverse treatment events to ensure long-term quality of life in NSCLC survivors.

Before the results of this study can be translated into clinical practice, further research is warranted to address the limitations of this study. Dose-volume parameters for other cardiac substructures should be investigated, and a cutoff value should be determined to establish constraints that can be applied in clinical practice. Consequently, an external validation of the findings should be performed. If using automated contouring models for other structures, such models should be validated and clinically approved. While adjustments were made for confounders such as age, sex, pre-RT Hgb levels and pre-existing arterial hypertension, other potential confounders were not accounted for, including cardiotoxicity from systemic treatment, as nearly all patients received concomitant therapy. Given the observed associations, we suggest that clinicians consider documenting and reviewing high-dose parameters to RA, SAN and other cardiac substructures. While no threshold for dose modification can yet be defined, awareness of substructure doses and clinical monitoring in high-exposure cases may help identify patients at risk. Thus, a prospective study could be a possible future research approach.

## Conclusion

This study revealed the association between radiation exposure to RA, SAN, MHD and V_40Gy_ and the risk of DNAF and DNHD in NSCLC patients. These findings indicate that RA and SAN may be considered organs at risk in future RT planning of NSCLC patients, and the current practice of heart delineation as an organ at risk remains crucial in reducing the incidence of DNAF and DNHD.

## Supplementary Material



## Data Availability

Data will be made available by the author upon request. All authors contributed to and approved of the final manuscript.
