# Supplementary material for: Association of right atrium and sinoatrial node irradiation with atrial fibrillation and radiation-induced heart disease in non-small cell lung cancer
Source: Acta Oncol. 2026 Feb 6;65:43885. doi: 10.2340/1651-226X.2026.43885 (PMC12887752; doi:10.2340/1651-226X.2026.43885)
Supplement: Supplementary file 1 [file AO-65-43885-s1.pdf]

Supplementary material has been published as submitted. It has not been copyedited, or typeset by Acta Oncologica

Supplementary material:

Table S1: Survival in follow-up

**Survival in follow-up**	n (%)
All-cause mortality	203 (82.2)
Progression of disease lung cancer	119 (48.2)
Infection or sepsis	38 (15.4)
Sudden cardiac death	6 (2.4)
Other cardiac condition; AFLI, AMI etc.	10 (4)
Radiation pneumonitis	5 (2)
Other pulmonary condition; COPD, etc.	12 (4.9)
Other or unknown	21 (8.5)
Median overall survival [median months post-RT (95% CI)]	27.4 (24.5 - 35.7)
Median survival with DNAF [median months post-RT (95% CI)]	3.9 (2.3 - 10.9)
Median survival with DNHD [median months post-RT (95% CI)]	4.9 (3.9 - 14.4)

**Survival in follow-up**	n (%)
All-cause mortality	203 (82.2)
Progression of disease lung cancer	119 (48.2)
Infection or sepsis	38 (15.4)
Sudden cardiac death	6 (2.4)
Other cardiac condition; AFLI, AMI etc.	10 (4)
Radiation pneumonitis	5 (2)
Other pulmonary condition; COPD, etc.	12 (4.9)
Other or unknown	21 (8.5)
Median overall survival [median months post-RT (95% CI)]	27.4 (24.5 - 35.7)
Median survival with DNAF [median months post-RT (95% CI)]	3.9 (2.3 - 10.9)
Median survival with DNHD [median months post-RT (95% CI)]	4.9 (3.9 - 14.4)
